# Treatment and Prevention of Bleeds in Haemophilia Patients with Inhibitors to Factor VIII/IX

**DOI:** 10.3390/jcm6040046

**Published:** 2017-04-17

**Authors:** Angiola Rocino, Massimo Franchini, Antonio Coppola

**Affiliations:** 1 Haemophilia and Thrombosis Centre, San Giovanni Bosco Hospital, Napoli 80144, Italy; 2 Department of Transfusion Medicine and Haematology, Carlo Poma Hospital, Mantova 46100, Italy; massimo.franchini@asst-mantova.it; 3 Regional Reference Centre for Coagulation Disorders, Federico II University Hospital, Napoli 80131, Italy; antocopp@unina.it

**Keywords:** bleeding, haemophilia, inhibitors, bypassing therapy, prophylaxis

## Abstract

The development of alloantibodies neutralising therapeutically administered factor (F) VIII/IX (inhibitors) is currently the most severe complication of the treatment of haemophilia. When persistent and at a high titre, inhibitors preclude the standard replacement treatment with FVIII/FIX concentrates, making patients’ management challenging. Indeed, the efficacy of bypassing agents, i.e., activated prothrombin complex concentrates (aPCC) and recombinant activated factor VII (rFVIIa), needed to overcome the haemostatic interference of the inhibitor, is not comparable to that of factor concentrates. In addition, the therapeutical response is unpredictable, with a relevant inter-individual and even intra-individual variability, and no laboratory assay is validated to monitor the efficacy and safety of the treatment. As a result, inhibitor patients have a worse joint status and quality of life compared to inhibitor-free subjects and the eradication of the inhibitor by immune tolerance induction is the preeminent therapeutic goal, particularly in children. However, over the last decades, treatment with bypassing agents has been optimised, allowing home treatment and the individualisation of regimens aimed at improving clinical outcomes. In this respect, a growing body of evidence supports the efficacy of prophylaxis with both bypassing agents in reducing bleeding rates and improving the quality of life, although the impact on long-term outcomes (in particular on preventing/reducing joint deterioration) is still unknown. This review offers an update on the current knowledge and practice of the use of bypassing agents in haemophiliacs with inhibitors, as well as on debated issues and unmet needs in this challenging setting.

## 1. Introduction 

**Figure 1:**
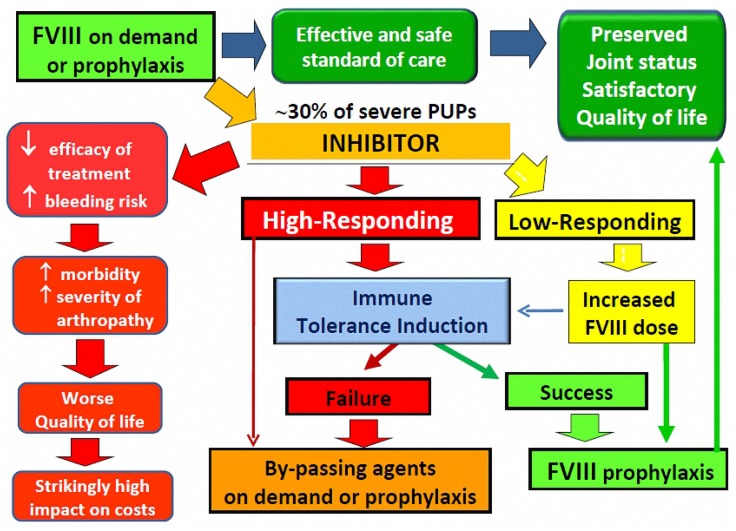
The administration of FVIII concentrates on-demand or, in particular, on long-term prophylaxis is the effective and safe standard of care in patients with severe hemophilia A, enabling to preserve joint status and a satisfactory quality of life. These achievements are precluded in approximately 30% of previously untreated patients (PUPs) who develop neutralizing alloantibodies (inhibitors), associated with reduced efficacy of treatment and increased bleeding risk, resulting in higher morbidity, severe joint deterioration and, in turn, worse quality of life and marked increase of healthcare costs. Inhibitor eradication through immune tolerance induction (ITI), able to restore FVIII replacement, is the primary objective of management of patients with high-responding inhibitors and of those with low-responding inhibitors in whom FVIII treatment is unfeasible in spite of increased doses. While awaiting inhibitor eradication and in patients failing ITI, patients with HR inhibitors are managed with bypassing agents given on-demand or on prophylaxis.

The development of alloantibodies (inhibitors) that neutralise the clotting activity of exogenous coagulation factor (F) given as replacement therapy is currently the most serious complication of the treatment of haemophilia. Inhibitors develop in approximately one-third of previously untreated patients (PUPs) with severe haemophilia A (i.e., patients with residual FVIII levels below 1 IU/dl), usually within the first 10–15 days of treatment with replacement factor concentrates [1]. The risk of inhibitor development then declines, becoming almost negligible in previously treated patients (PTPs) who have been exposed to FVIII concentrates for more than 50–150 days. However, the risk never disappears, persisting throughout one’s life, and shows a slight increase in the elderly [2]. The mechanisms responsible for inhibitor formation remain only partially understood, but studies in PUPs with severe haemophilia A have led to the identification of several risk factors, of both a genetic nature (null mutations in the *F8* gene, genotype of the major histocompatibility complex, polymorphisms of immunoregulatory genes, ethnicity) and those which are treatment-related, which indicate a multifactorial pathogenesis, resulting from complex interactions between genetic and environmental influences [3,4,5]. Inhibitors develop much more rarely in patients with a moderate (FVIII 1%–5%) or a mild form (FVIII > 5%–40%) of haemophilia A and, unlike that in those with severe haemophilia A, the risk of inhibitor formation has been found to increase in parallel with exposure to FVIII concentrates in these patients, so that inhibitors often develop during adulthood, frequently after a period of intensive treatment [6,7,8,9]. The incidence of inhibitors is also lower in PUPs with haemophilia B, in whom they are often associated with large deletions in the *F9* gene [10]. However, the management of patients with haemophilia B and inhibitors is further complicated by severe allergic reactions occurring in association with the administration of FIX-containing products in approximately half of patients [11]. The aetiology of such reactions is still unknown. On the basis of the highest documented inhibitor level and the occurrence of an anamnestic response after re-exposure to the factor concentrate, inhibitors are classified as low-responding (LR, at all times <5 BU/mL) or high-responding (HR, historical inhibitor peak >5 BU/mL at least once) [12]. Patients with LR inhibitors usually have fewer clinical problems because haemostasis can usually be ensured by saturating the inhibitor through the administration of higher doses of the deficient factor. By contrast, HR inhibitors rule out the use of standard on demand therapy and prophylaxis and, although bleeds are not more frequent than in patients without inhibitors [13], alternative haemostatic agents are required, which have poorer efficacy and safety profiles than factor concentrates. Bleeding episodes may, therefore, be much more difficult to control, resulting in higher risks of morbidity, mortality, and disability, with a profound effect on patients’ quality of life and healthcare costs [13,14,15]. For these reasons, the primary aim of treatment is the permanent eradication of the inhibitor by immune tolerance induction (ITI), enabling effective replacement therapy and making prophylaxis feasible, with the ultimate goal of preventing, or at least lessening, the negative impact that persistent inhibitors have on patients’ morbidity and quality of life (Figure 1). ITI treatment is recommended by international and national guidelines [16,17,18,19,20], the European principles of haemophilia care [21], and expert panels [22,23,24] for all patients with severe haemophilia A and HR inhibitors. Children with recent onset HR inhibitors are the main candidates, because early eradication can optimise the cost-utility ratio in a long-term perspective [25,26,27]. ITI should also be considered for patients in whom persistent LR inhibitors interfere with standard-dose prophylaxis or on-demand treatment [22,23,24]. However, ITI fails in about one third of patients; it takes a long time to be achieved in a substantial proportion of cases, and its availability is restricted in many areas due to its high cost. ITI appears to be less effective in eradicating inhibitors in patients with haemophilia B [28,29,30], and data from the International Society of Thrombosis and Haemostasis (ISTH) Registry show that 38% of patients suffered nephrotic syndrome during ITI [29]. International groups of experts do not, therefore, recommend ITI for patients with haemophilia B with inhibitors [16,17,18,19,20,21,22,23,24]. However, data recently collected in Italy demonstrated the complete success of inhibitor eradication in four of five patients with severe haemophilia B with inhibitors treated with a low-dose ITI regimen [31]. Data on ITI in patients with non-severe haemophilia A are mostly anecdotal, and strong evidence supporting the systematic performance of ITI in such patients is therefore lacking [17,18,30,32]. However, a case series of 32 patients with mild haemophilia A demonstrated a potential effect of rituximab, an anti-CD20 monoclonal antibody, in eradicating inhibitors [33]. Furthermore, a recent report from the INSIGHT study group, on the largest retrospective cohort of 101 patients with non-severe haemophilia A, described inhibitor disappearance after eradication treatment in 21/28 (75%) patients, using widely variable strategies, including both ITI and immunosuppression [34]. Nevertheless, most clinicians consider implementing ITI in patients with frequent bleeding, although there is no evidence in the literature supporting the choice of a specific ITI protocol [17,18,30,32].

The so-called “bypassing agents” are currently the treatment of choice for the management of bleeding in patients with actual high-titre inhibitors. Selecting the best treatment strategy by the use of these agents is of fundamental importance both for children waiting for ITI, in order to prevent the irreversible consequences of difficult-to-treat haemorrhages, and for patients who are not eligible for or who have failed to benefit from ITI, in order to lessen bleeding-related morbidity and improve the quality of life. Over the last decade, advances have been made in the management of bleeding with bypassing agents and prophylactic regimens are being increasingly used. This review offers an update on the current state-of-the-art knowledge and practice of the use of bypassing agents in haemophiliacs with inhibitors, and discusses current issues and unmet needs from the authors’ personal perspective.

## 2. Treatment of Acute Bleeding

The treatment of acute bleeding in patients with an inhibitor should be tailored to the type and severity, the actual inhibitor level, and the anamnestic response (LR or HR after exposure to the missing factor). Replacement treatment with FVIII or FIX concentrate is the ideal haemostatic approach [17,18], and still applies in the case of LR inhibitors which are commonly overcome with higher factor doses. The dose necessary to neutralise the inhibitor and increase the amount of FVIII or FIX to a haemostatic level varies widely and several dosing algorithms have been proposed [35,36]. The initial bolus must provide enough FVIII or FIX to neutralise circulating inhibitors, as well as the amount required to increase the clotting factor to haemostatic levels. Treatment is then continued with bolus infusions capable of maintaining target FVIII/FIX levels until bleeding is controlled. Alternatively, a continuous infusion can be used, with the infusion rate adjusted on the basis of the circulating levels reached. However, given the variability of the response, patients treated with FVIII/FIX should be monitored regularly (at least daily) with assays of FVIII/FIX levels, to detect any anamnestic increase in inhibitor activity [17,18]. The same approach can be reserved to the management of severe bleeds in patients with HR inhibitors but actual low levels (<5 BU/mL), before the onset of the anamnestic response. In patients with high inhibitor levels (>5 BU/mL) at the time of bleeding, bypassing agents constitute the only available strategy for the treatment of the bleeding and the prevention of further episodes. Currently available options of bypassing agents are an activated prothrombin complex concentrate (aPCC; FEIBA^®^, FVIII inhibitor bypassing activity; Baxalta part of Shire, Vienna, Austria) and recombinant activated FVII (rFVIIa; NovoSeven^®^, Novo Nordisk A/S, Bagsvaerd, Denmark). Plasmaderived porcine FVIII was previously widely used in this setting, but is no longer available; however, a recombinant porcine FVIII has been developed [37] and is currently in clinical trials for use in congenital haemophilia A with inhibitors. The characteristics and licensed regimens of treatment with aPCC and rFVIIa are summarised in Table 1.

rFVIIa is produced by baby hamster kidney (BHK) cells that express the cloned human *F7* gene. A new formulation of rFVIIa includes sucrose and L-methionine, so that the product can be stored at room temperature before reconstitution. rFVIIa promotes haemostasis by activating FX directly on the platelet surface, thereby bypassing the tenase complex [38]. The consequent increase in thrombin generation enhances platelet aggregation, leads to the full activation of thrombin-activatable fibrinolysis inhibitor and FXIII, and ensures that a tight fibrin plug is produced [39]. The half-life of rFVIIa is 2.3 h in adults, although it is potentially shorter in children [40].

FEIBA is a vapour-heated concentrate of plasma-derived, vitamin K-dependent clotting factors (FII, FVII, FIX, and FX) in both zymogen and active forms. Its mechanism of action is multifactorial, although FII and activated FX are considered to be the most important components [41]. Clinical studies, mostly retrospective, have demonstrated that both agents are effective, achieving haemostasis in more than 90% of surgical interventions, in about 80% of acute bleeding episodes, and during extensive use in clinical practice [42,43], even in the setting of home treatment [44,45,46]. As regards rFVIIa, randomised clinical trials have demonstrated the comparable efficacy and safety of a single high-dose (270 µg/kg) versus repeated standard doses (90 µg/kg) [47,48]. Thus, the high-dose regimen may be conveniently used, especially in children with venous access problems. However, some patients may respond better to rFVIIa or aPCC and, importantly, the same patient may have different responses to one product or to the other on different occasions, even when the bleeding episodes are similar. Few studies have compared the efficacy of aPCC versus rFVIIa, but one head-to-head, prospective, open-label, randomised, crossover trial (the FEIBA NovoSeven comparative study, FENOC) of 48 inhibitor patients confirmed such a variability [49]. The two treatments (a single dose of FEIBA^®^, 75–100 U/kg, or two doses of rFVIIa, 90–120 μg/kg), used alternatively in two joint bleeding episodes, showed a substantially similar efficacy (approximately 80%) in the management of early haemarthroses, although the statistical requirements for equivalence were not met. However, 10%–44% of the individuals responded more favourably to one or the other of the products 6 and 12 h after treatment. In another head-to-head, randomised controlled trial, by Young et al., the efficacy of one dose of aPCC 75 U/kg was compared to that of three infusions of rFVIIa 90 μg/kg (at 0, 3, and 6 h) or a single bolus of rFVIIa 270 μg/kg in 27 hemophilia patients with inhibitors [50]. The efficacy was assessed using a global response algorithm that took into account pain and mobility scores at 9 h after the start of treatment and the requirement for additional haemostatic agents (rescue medication) within the 9-h period of observation. No statistically significant differences were found in the global algorithm or in the pain and mobility scores measured separately. Although rescue medication was required by a significantly lower percentage of patients in the rFVIIa 270-μg/kg group than in the aPCC group, there was not a statistically significant difference in the rescue medication use between the aPCC group and the rFVIIa 3 × 90-μg/kg group. Overall, on the basis of the results of these two randomised controlled trials, a Cochrane Collaboration review concluded that there was no compelling evidence for the superior efficacy of one product over that of the other in the management of haemarthroses [43]. Studies on other types of bleeds have not been performed. However, some clinicians prefer to use rFVIIa in children because of its recombinant origin and because it does not contain traces of FVIII or FIX, which could potentially induce an anamnestic response in candidates for ITI. This effect has been reported to occur in 22% to 31.5% patients with haemophilia A receiving the aPCC [51,52]. However, the anamnestic response is usually transient, and does not affect the efficacy of the aPCC treatment. Other elements that may influence the choice between the two products include the infusion characteristics (larger volumes but less frequent infusions with aPCC) and, in haemophilia B patients, the avoidance of allergic reactions and anamnesis of the inhibitor, which can occur with aPCC use, because of the presence of FIX. Moreover, given the inter- and intra-individual variability of the haemostatic response to bypassing agents, an unsatisfactory response should result in an early change of the treatment strategy, by intensifying the regimen (increasing the dose and/or the frequency of the agent being used), or by switching the type of bypassing agent, rather than prolonging the use of the same product and dosing [53]. In addition, it should be taken into consideration that bleeds unresponsive to intensive treatment with one or the other of the two bypassing agents used singly have been shown to be controlled by their use in combination or sequentially (at short intervals) [54]. In fact, the two products have been reported to have a synergistic effect in vitro [55] and in vivo [56]. Eleven sequential courses of bypassing therapy were recorded in a European survey of nine haemophiliacs, aged between 9 and 73 years old (median 24), whose bleeding, in five cases due to major surgery, was unresponsive to single therapy with one of the two bypassing agents [57]. Both bypassing agents were administered, alternating one aPCC dose (20–80 U/kg every 8–12 h) with one to three rFVIIa doses (90–270 μg/kg every 3–12 h). Bleeding was successfully controlled within 12–24 h in all patients and treatment was discontinued after 1–15 days. No clinical adverse events were noted, but D-dimer levels increased significantly in three of the five patients in whom this parameter was assessed. Combination therapy with bypassing agents may, therefore, predispose to thrombotic complications and must only be used, after an appropriate risk-benefit assessment, as salvage therapy for a short period of time in a hospital setting, when other interventions have failed [17,18].

As regards the treatment of haemophilia B patients with a history of severe reactions to FIX-containing concentrates, it is generally advised that their bleeds be treated with rFVIIa [17,18,29,58]. In patients with mild and moderate haemophilia A who develop inhibitors, the risk of bleeding may be increased as a result of the cross-reactivity between the inhibitor and the native FVIII synthesised by the patient, which can reduce circulating FVIII levels to <1 IU/dL [59]. In order to minimise the exposure to FVIII concentrates, the use of desmopressin (DDAVP) is encouraged in patients who successfully respond to such treatment [18]. Inhibitors may disappear spontaneously if no further treatment with FVIII concentrates is administered, but this does not exclude inhibitor relapse after re-exposure to FVIII. DDAVP may be an option for patients who have circulating endogenous FVIII levels. However, these patients should have a DDAVP trial with FVIII levels measured before and 1 and 4 h after administration of the product: if the response is adequate, DDVAP, in combination with tranexamic acid, should be used for the treatment of bleeds likely to respond [17]. In patients unresponsive to this drug and those who are not candidates for FVIII replacement therapy, bleeding episodes can be treated or prevented with bypassing agents [17,18,32], although some clinicians prefer to use rFVIIa, with the aim of avoiding anamnestic responses to further exposure to FVIII.

Regarding safety, the administration of activated factor concentrates in patients with inhibitors has been associated, albeit rarely, with thrombotic complications, including deep vein thrombosis, pulmonary embolism, disseminated intravascular coagulation, and myocardial infarction [60,61,62,63,64]. It is believed that the thrombotic risk is related to the dose of the bypassing agents used, the duration of treatment, and the combination of other risk factors, such as hepatic, cardiovascular, and metabolic disorders, prolonged bed-rest, active infections, and surgery which increases the likelihood of patients developing thrombotic events. The concomitant use of antifibrinolytics and thromboprophylaxis is still under discussion in this setting. The rationale for the concomitant use of bypassing agents and tranexamic acid is to exploit a potential synergistic effect and increase clot stability [65]. Tranexamic acid is generally used in association with rFVIIa, whereas it was not often used with aPCC in the past, because of concerns about the risk of thromboembolic complications. However, a recent literature review of studies and individual case reports showed that the concomitant use of aPCC and tranexamic acid during dental procedures, orthopaedic surgery, gastrointestinal bleeding, epistaxis, and cerebral haemorrhages was safe, well-tolerated, and effective, and no thrombotic complication was reported [66]. Nevertheless, additional randomised controlled studies are warranted in order to confirm these findings. Thus, currently, tranexamic acid should be considered in all patients with inhibitors, irrespective of the bypassing agents used, but should be used with caution in association with aPCC. The association of tranexamic acid is especially useful for the management of mucosal bleeds [17,58].

Local measures, such as thrombin or fibrin glue, may improve haemostasis and should be considered in patients with persisting bleeding complications that may be controlled by the use of such adjunctive therapy [67]. Finally, regarding the use, still debated, of thromboprophylaxis during treatment with bypassing agents, this strategy could be considered in patients who receive concomitant intensive treatment with bypassing agents during ITI when the inhibitor has been reduced to low levels, but not completely eradicated, especially in patients with comorbidities or risk factors associated with a high risk of thrombotic complications.

## 3. Monitoring Therapeutic Efficacy of Haemostatic Treatment

No laboratory assay is currently validated either to monitor the efficacy of bypassing agents, or to determine their optimal dose. Both thromboelastography (TEG) and the thrombin generation assay (TGA) may offer some information relevant for the treatment of individual subjects [68,69]. These laboratory tests have shown that ex vivo responses to both bypassing agents are dose-dependent. However, so far, no routine laboratory test has been found suitable for monitoring the efficacy and safety of these drugs in routine clinical practice [70,71]. Moreover, the well-recognised, relevant variability in the clinical phenotype among patients with severe haemophilia, as well as in the clinical responses to the bypassing agents, is reflected by variations in the results of in vitro assessments of haemostatic interventions. Given this scenario, the choice of which of the bypassing agents to use is mainly based on the individual’s clinical response, with the feasibility of home treatment also taken into consideration. Such a treatment approach offers the possibility of an early intervention, but clinicians and patients should appreciate that in order to obtain the best response from treatment with bypassing agents, the bleeding must be recognized early and the treatment, which should be adequate in terms of dosing, timing, and number of infusions, needs to be initiated promptly. Moreover, such an approach requires that patients and their caregivers are educated very thoroughly on how to evaluate the efficacy of the treatment and recognize the onset of adverse events [42]. In this respect, clinical assessment remains the primary method for determining the need for additional doses or switching to the alternative bypassing agent. Teitel et al. developed a consensus algorithm intended to help physicians to optimise the timing of treatment decisions, since this could result in faster responses and better outcomes of problem bleeds in patients with severe haemophilia A and inhibitors [53]. Problem bleeds were defined as bleeding episodes that did not respond to initial therapy with a single bypassing agent within a reasonable amount of time, defined as 2–4 h for life-threatening bleeds and 8–12 h for bleeds that were not life-threatening. The algorithm provided a guide to the management of difficult-to-treat bleeds, and highlighted the need to change treatment at early signs of an inadequate haemostatic response, thus preventing extensive treatment that may lead to suboptimal outcomes and unnecessary costs. Berntorp et al. provided a consensus definition for non-life-threatening joint and muscle bleeds that are unresponsive to bypassing agents [72]. A non-responsive bleeding episode was identified on the basis of persistent or worsening pain, increased or unchanged swelling/tension, decreased or unchanged mobility relative to a bleed-specific baseline, the patient’s perception of an active bleed, laboratory parameters such as haemoglobin levels, and the TGA. It was suggested that a non-life- or limb-threatening joint or muscle bleed should be considered unresponsive if the specified criteria are met 24 h after treatment initiation. The criteria can be assessed subjectively by the patient/parent and/or objectively by the clinician. Although the suggested criteria need to be validated in a real-world scenario, they should be considered a methodology to guide the recognition of unresponsive bleeds and the change of treatment as soon as an inadequate response is observed.

## 4. Bleeding Prevention

The standard of care for patients with severe haemophilia without inhibitors is primary prophylaxis, aimed at preventing any joint bleeding and deterioration of the joint status and at enabling patients to gain an optimal quality of life [16,21]. Secondary and tertiary prophylaxis may decrease and possibly stop recurrent haemarthroses, prevent the involvement of new joints, arrest, or at least delay, the progression of joint damage, improve the quality of life, and lower the risk of other serious bleeds, such as intracranial haemorrhage [73]. The goals should be the same for haemophilia patients with inhibitors, who have a greater morbidity rate and impaired quality of life. In the past, a lack of literature data, and the short half-life of bypassing agents and their high cost have limited the use of prophylaxis in the subset of haemophilia patients with inhibitors. However, growing evidence on the efficacy of prophylaxis at reducing the number of bleeds and improving the quality of life is encouraging the expansion of this management strategy to also include inhibitor patients. In a meta-analysis of six studies (involving a total of 34 inhibitor patients) on prophylaxis with aPCC, Valentino [74] found a 64% reduction of the bleeding rate in 31 of the 33 evaluable patients (94%), independent of the type of bleeding. In the three studies that specifically considered all joint bleeding, the annual joint bleeding rate decreased by an average of 74% in 18 evaluable patients while these subjects were on prophylaxis. The mean prophylactic dose was 78.5 U/Kg and the infusions were usually administered three or four times weekly. The reduced bleeding rate was accompanied by an improved quality of life. However, this prophylactic regimen did not prevent or halt joint deterioration in patients with significant arthropathy before starting the treatment, although it did appear to be useful for preventing joint damage in previously unaffected joints. In the FEIBA Post-Authorization Safety Surveillance (PASS) study, intended to collect real-world data on the safety and effectiveness of prophylactic or on-demand treatment with FEIBA^®^ in patients with congenital or acquired haemophilia A, too few patients switched from on-demand to prophylaxis during the observation period to enable a decrease in bleeding frequency during prophylaxis to be evaluated; however, the proportion of patients who did not have any bleeds was higher among patients on prophylaxis than among those being treated on demand (24.5% versus 7.0%, respectively), with a median number of 5.0 bleeds per year (range, 0.0–55) in patients on prophylaxis receiving a median of 76.6 U/Kg per day [62]. In the retrospective, observational PRO–PACT study of 86 inhibitor patients receiving secondary prophylaxis with rFVIIa, the percentage reduction of bleeding (primary study outcome measure) was 46% (95% CI, −54.0 to −38.2) in patients who had at least one bleeding episode before starting prophylaxis and 52% (95% CI, −60.7 to −43.3) in patients who had at least one bleeding episode per month during the six months prior to the study [75]. No adverse events were reported. Furthermore, there were fewer hospital admissions and the time spent in hospital was shorter. The use of secondary prophylaxis with bypassing agents in haemophiliacs with inhibitors has been studied in three randomised trials: one with rFVIIa and two with aPCC [76,77,78]. The patients’ characteristics and main study findings are reported in Table 2. In a multicentre, randomised, parallel group study, 22 subjects with haemophilia A and an HR inhibitor being treated with a bypassing agent were randomised to receive rFVIIa 90 or 270 μg/kg/day for three months [76]. The rate of bleeding during the pre-prophylaxis period was 5.6 and 5.3 bleeds per month in the two groups, respectively. Over the three months of prophylaxis, this decreased to 3.0 bleeds per month in the 90 μg/kg group and 2.2 bleeds per month in the 270 μg/kg group, with corresponding reductions in target joint bleeds of 43% and 61%, respectively. No significant differences were detected between the two dose regimens but, interestingly, most of the reductions in the bleeding rate were maintained over a three-month observation period after prophylaxis. The quality of life also improved, although not to a statistically significant extent, hospital admissions were reduced, and fewer days were missed from school or work [79]. As regards the other bypassing agent, two randomised trials showed that prophylaxis with aPCC significantly reduced the total, joint, and target joint bleeding episodes, and significantly improved patients’ quality of life compared with on-demand treatment [77,78,80]. The reductions in the bleeding rate recorded in these studies ranged from 45% to 72.5%, with a 43%–75% reduction in episodes of target joint bleeding in patients on secondary prophylaxis. In the PROOF study, new target joints occurred substantially less frequently in the group treated with prophylaxis than in that receiving on-demand treatment: there were seven new target joints in five (29.4%) out of 17 patients on prophylaxis (range per patient: 1–2) versus 23 new target joints in 11 (57.9%) out of 19 patients being treated on demand (range per patient: 1–6) [78]. Moreover, in the same study, 12 of 16 (75%) individuals receiving prophylaxis were considered good responders (defined as a ≥50% reduction in the bleeding rate), compared to only two of 19 (10%) individuals being given on-demand therapy. Two of 17 subjects on prophylaxis did not have any bleeds during the study period. This compares with 16 of 26 patients (62%) who were good responders in the Pro-FEIBA study, six of whom had no bleeds [77]. The quality of life was also improved in subjects receiving prophylaxis in all three studies, but the difference was only statistically significant for the subjects included in the rFVIIa study and for the good responders in the Pro-FEIBA study [79,80]. Overall, in the light of these studies, prophylaxis with bypassing agents is being increasingly mentioned in expert recommendations and guidelines [17,18,20,23,81,82], particularly for those subjects with frequent or severe bleeding (e.g., intracranial haemorrhage). More data should, however, be collected in order to identify the profile of patients who could obtain the greatest benefit from such an approach, as well as to assess the long-term outcomes in terms of both the efficacy (bleeding reduction and prevention of late-stage joint damage) and safety of prophylaxis regimens, and to perform reliable cost-effectiveness and cost-utility analyses.

## 5. Unresolved Issues and Unmet Needs

The development of the two currently available bypassing agents has greatly bettered the management of haemophiliacs with inhibitors, enabling home treatment with a notable improvement of patients’ quality of life. Overall, the currently published data indicate a substantial equivalence of the two products in terms of the efficacy and occurrence of adverse thrombotic events. However, only a few elderly patients have been included in clinical studies and definitive conclusions cannot be drawn on the safety of bypassing agents in these patients, especially if treated with prophylactic regimens. Moreover, the management of inhibitor patients still has a series of unsolved issues. These include the 10%–20% of bleeds that are not satisfactorily resolved, the variability and, often, unpredictability of the responses, the absence of validated laboratory tests to monitor the efficacy and safety of treatment, and the potential benefits of individualised prophylactic regimens. The three available randomised studies (Table 2) provide strong evidence that prophylaxis improves care for inhibitor patients. However, prophylactic treatment in patients with inhibitors is not as effective as that in patients without inhibitors. This further confirms the need to eradicate the inhibitor by ITI as early as possible, because it enables the resumption of replacement therapy, particularly of standard prophylactic regimens in order to avoid/reduce bleeding-related joint damage. In addition, data from the literature support the use of prophylaxis with bypassing agents during ITI, as implemented in the ITI Bonn protocol [83]. This approach has been recommended by expert groups [22,23,24,30,81,82], but still needs to be further personalised on the basis of the patient’s clinical phenotype. However, whether the bleeding phenotype of subjects with inhibitors differs from that of those without inhibitors, is not yet clear. This issue is difficult to investigate retrospectively, because the outcomes which are usually considered are the bleeding frequency and the efficacy of treatment, both for the management of acute bleeding episodes and for the prevention of recurrent bleeds. However, such characteristics vary greatly, even within the same individual, the clinical response to bypassing agents is poorly predictable, and the overall success rates that can be achieved with these agents do not reach those afforded by factor replacement therapy in patients without inhibitors. In the COCIS study, interestingly, inhibitor patients, almost all high responders, were reported to have an average bleeding frequency of 0.6 events per patient per month, with 19% not have any bleeding at all [13]. This bleeding frequency does not differ dramatically from that observed in other cohorts of adults with severe haemophilia without inhibitors being treated on demand. A possible explanation for this observation could be a particularly judicious behaviour in performing daily activities, either because of the fear of bleeding or because of severely impaired motion. The orthopaedic status of individuals with inhibitors is worse than that of age-matched patients without inhibitors [14]. The European Study on Orthopaedic Status (ESOS) did not detect any difference in the annual frequency of muscle bleeds in patients with severe haemophilia A or B among two cohorts, aged 14–35 or 36–65 years, with inhibitors and one cohort, aged 14–35 years, without a history of inhibitors, whereas the frequency of joint bleeds in the young cohort with inhibitors was similar to that in the age-matched controls, but double that in the older inhibitor cohort. Likewise, clinical and radiological orthopaedic scores were worse in the cohorts with inhibitors than in the age-matched group of patients without inhibitors. In addition, subjects with inhibitors were admitted to hospital for orthopaedic procedures more frequently than those without inhibitors, regardless of age, and walking aids were needed more frequently among inhibitor patients. On the whole, the results of this study suggest that end-stage joint damage, rather than the presence of inhibitors *per se*, may influence the bleeding phenotype in the long term. Prospective studies comparing the bleeding phenotype of patients with and without inhibitors have not been published so far. However, defining the bleeding phenotype in patients with inhibitors is clinically important, because it may have a strong influence on treatment strategies, particularly in children who are candidates for bypassing agent prophylaxis prior to and during ITI. In this respect, some authors have proposed the early implementation of prophylaxis in children with recent-onset inhibitors, with the aim of the “primary” prevention of arthropathy, similar to the approach in non-inhibitor children [84]. However, few data, limited to the short-term, are available and, as a whole, it is still unknown whether and to what extent the reduction of the bleeding rate of 45%–74%, observed in the published clinical trials of “secondary/tertiary” prophylaxis with bypassing agents, can significantly reduce/prevent joint deterioration. In addition, the issue of subclinical joint bleeding is an object of debate. The role of unrecognised, subclinical joint bleeds was hypothesised in the US Joint Outcome Study, in which 65 children <30 months old with severe haemophilia A were randomised to FVIII prophylaxis or enhanced episodic therapy, and followed up for five years [85]. The number of bleeding episodes was strongly correlated with joint damage, as determined by a magnetic resonance imaging (MRI) score, but not in all subjects. Indeed, MRI demonstrated joint damage in some of the paediatric patients who had no clinical evidence of joint bleeds. Other reports have confirmed this observation [86,87]. Moreover, the occurrence of subclinical bleeding appears to be confirmed by histological and clinical observations and may contribute to the development of arthropathy [88,89]. The issue of subclinical bleeds is complex, because besides the current inability to detect them simply, strategies for their treatment and prevention are lacking. In order to improve our knowledge of these aspects, joint ultrasonography appears to be a promising approach [90] and, in the future, the identification and validation of biochemical markers of progressing joint disease could be useful to guide treatment [91]. However, the ‘acceptable’ number of joint bleeds may differ among patients because of inter-individual variation in the inflammatory response to bleeding. Such variation could be the result of genetic polymorphisms that modify synovial or vascular cell proliferation; neutrophil or monocyte infiltration into the joint space, osteoblast, or osteoclast activation; and possibly, chondrocyte function [92]. Furthermore, an acceptable bleeding rate may depend on the overall individual therapeutic objective. In children, the major aim should be to prevent any haemarthroses in order to preserve joint health, whereas in adults it could be to decrease the number of bleeds/target joints in order to improve the quality of life and joint status. The same paradigm should apply to patients with and without inhibitors. However, these clinical aims are more difficult to achieve in patients with inhibitors, because of the lower efficacy of bypassing agents compared with FVIII/FIX replacement. For this reason, the eradication of the inhibitor remains a priority. However, the efficacy of prophylaxis with bypassing agents may, possibly, be improved by tailoring the individual regimen by a frequent and regular assessment of the outcome measures, such as the physical, functional, radiological, and health-related quality of life. The type of bypassing agent, the dose, and its frequency of administration should be adjusted to find a prophylactic regimen that is clinically effective, cost-effective, and practical. As for non-inhibitor patients, of course, a single approach will not be appropriate for all individuals [93], whereas individualised prophylaxis should increase the probability that the goal of minimal or no bleeding is achieved, at least in children exhibiting a recent onset of an inhibitor, without increasing overall healthcare costs. The issue of the costs of inhibitor patients, being 3–15 times higher than those of non-inhibitor ones [13,94,95], is even more relevant given the current scenario of constraints on healthcare expenditure. In addition, patients should be managed by a multidisciplinary team and be involved in the decision-making, so they must be aware of the importance of every bleed and the need to strictly adhere to the prescribed treatment regimen.

## 6. Concluding Remarks

Inhibitor development is a challenging complication of haemophilia treatment. Great progress has been made in the last decades, including the identification of patients at a high risk of inhibitor development, the identification of some predictors of successful inhibitor eradication, the improvement of treatment, and the prevention of bleeding episodes. However, the current scenario indicates that further progress may be achieved through a better understanding of how to individualise treatment with the presently available bypassing agents. Major efforts need to be devoted to the education of patients and their caregivers in the perspective of early treatment of any bleed, thus avoiding the negative effects of bleeding on patients’ quality of life. In addition, the crucial clinical objective should be to identify tailored prophylactic regimens that take into account the bleeding phenotype, the individual response to one or other of the currently available bypassing agents, and adherence to the prescribed regimen. Further research is also needed to determine the economic impact of prolonged prophylaxis, as well as its long-term outcomes and potential benefits. These advances and perspectives mainly apply to the management of patients with haemophilia A and inhibitors, whereas knowledge and clinical experience remain limited for those with haemophilia B, in whom the occurrence of allergic reactions is a major challenge. Concerted research efforts and continuous clinical vigilance in the frame of large, long-term, prospective studies are needed to address the debated issues and unmet needs in this setting.

## Figures and Tables

**Table 1:** Characteristics and licensed regimens of bypassing agents for the treatment of patients with HR inhibitors.

Bypassing Agent	Activated Prothrombin Complex Concentrate (aPCC)	Recombinant Activated Factor VII (rFVIIa)
Source	Pooled human plasma	Recombinant coagulation FVIIa produced in baby hamster kidney (BHK) cells by recombinant DNA technology (Eptacog alfa)
Therapeutic indications	Treatment of spontaneous bleeding and cover of surgical interventions in: haemophilia A patients with inhibitors to FVIII patients with acquired inhibitors to FVIII prophylaxis in haemophilia A patients with HR inhibitors and frequent joint bleeding.	Treatment of bleeding episodes and cover of surgical interventions in: - patients with congenital haemophilia with inhibitors to FVIII or FIX >5 BU - patients with congenital haemophilia who are expected to have a high anamnestic response to FVIII or FIX administration - patients with acquired haemophilia.
Recommended regimen(s)	50 to 100 U*/kg Dose interval: 6–12 h. A single dose of 100 U/kg and a daily dose of 200 U/kg should not be exceeded unless the severity of bleeding warrants and justifies the use of higher doses. For prevention of bleeding episodes during prophylaxis, 70 to 100 U/kg every other day with dose adjusted based on the patient’s clinical response.	Initial dose: 90 μg/kg Dose interval: 2–3 h until haemostasis is achieved. If continued therapy is needed, the dose interval can be increased successively to every 4, 6, 8 or 12 h for as long as treatment is judged as being indicated. For mild to moderate bleeding episodes (including home therapy) two dosing regimens can be recommended: (1) Two to three injections of 90 μg/kg at 3-h intervals. If further treatment is required, one additional dose of 90 μg/kg (2) One single injection of 270 μg/kg. The duration of home therapy should not exceed 24 h.
Storage requirements	Room temperature (up to 25 °C) for up to 2 years.	Room temperature (up to 25 °C) for up to 2 years.
Volume	Diluent volume: 20 mL.	Diluent volume: 1.1 mL (1 mg); 2.1 mL (2 mg); 5.2 mL (5 mg).
Time of administration	The rate of intravenous administration should ensure the comfort of the patient and should not exceed a maximum of 2 U/kg per minute.	Intravenous bolus injection over 2–5 min
Half-life in plasma	Varying half-lives for the single components (FII, FIX, FX, FVIIa)	2.3 h (range 1.7–2.7)
Inhibitor anamnesis	Possible in about 20%–30% of patients, since FVIII coagulant antigen (FVIII C:Ag) is present in a concentration of up to 0.1 U/1 U of aPCC. Upon continued administration, inhibitors may decrease over time.	No
Association with antifibrinolytic agents	Systemic antifibrinolytics should be administered at least 6 h apart.	Commonly used.
Laboratory monitoring	Not standardised	Not standardised
Thrombotic risk	Yes	Yes

* One unit is defined as that amount of the product that shortens the activated partial thromboplastin time of a high titre FVIII inhibitor reference plasma to 50% of the blank value.

**Table 2:** Design, patients’ characteristics, and main findings of the prospective randomised studies on prophylaxis in inhibitor patients.

Bypassing Agent	rFVIIa		aPCC	
Study	Konkle et al. 2007 [76]		Lessinger et al. 2011 [77] PRO-FEIBA	Antunes et al. 2014 [78] PROOF
Design	Double-blind, parallel group trial		Open-label, cross-over trial (intra-individual comparison)	Open-label, two arm, parallel trial (inter-individual comparison)
Patients, *n*	22		26	36
Treatment	90 μg/kg/day vs. 270 μg/kg/day		85 ± 15 U/kg three times per week vs. on-demand treatment	85 ± 15 U/kg each other day (*n* = 17) vs. on-demand (*n* = 19)
Follow-up	3 months (vs. 3 months pre-study and 3 months post-prophylaxis)		6 months in each treatment regimen (3 months wash-out)	12 months
Age, years (range)	15.7 (5.1–56.5)		28.7 (2.8–62.8)	23.5 (7–56)
Bleeding rate (as inclusion criterion)	≥12 in 3 months		≥6 in 6 months	≥12 in 12 months
	90 μg/kg/day	270 μg/kg/day		
Mean bleeding rate on demand	5.6 *	5.3 *	13.1 ^	28.7 ^#^
Bleeding rate on prophylaxis	3.0 *	2.2 *	5.0 ^	7.9 ^#^
Reduction (%) on prophylaxis	45%	59%	62%	72.5%
Reduction (%) of target joint bleeds on prophylaxis	43%	61%	72%	75%
Other findings	Benefits extended during the 3 months after prophylaxis		62% good responders (bleeding rate reduction >50%, overall 84%). In 23% 0 bleeds	Lower development of new target joints on prophylaxis. 26% additional reduction of bleeding rate in the last 6 months vs. first 6 months

* Mean, per month; ^ mean, over 6 months; ^#^ Annualised bleeding rate.
